# Infrared spectral library of tooth enamel from African ungulates for accurate electron spin resonance dating

**DOI:** 10.1038/s41597-024-03725-y

**Published:** 2024-08-15

**Authors:** Michael B. Toffolo, Maïlys Richard

**Affiliations:** 1 Geochronology and Geology Programme, National Research Centre on Human Evolution (CENIEH), 09002 Burgos, Spain; 2 Archéosciences Bordeaux, UMR 6034 CNRS-Bordeaux Montaigne University, 33607 Pessac, France; 3 Department of Early Prehistory and Quaternary Ecology, University of Tübingen, 72070 Tübingen, Germany

**Keywords:** Palaeoecology, Biomineralization, Geochemistry, Characterization and analytical techniques, Palaeontology

## Abstract

Electron spin resonance coupled with uranium-series dating (ESR/U-series) of carbonate hydroxyapatite in tooth enamel is the main technique used to obtain age determinations from Pleistocene fossils beyond the range of radiocarbon dating. This chronological information allows to better understand diachronic change in the palaeontological record, especially with regard to the evolution of the genus *Homo*. Given the relative paucity of human teeth at palaeontological and archaeological localities, ESR/U-series is widely applied to the teeth of ungulate species. However, the accuracy of ESR/U-series ages is greatly affected by the incorporation of uranium in the enamel during burial in sediments. It has been shown that uranium content is positively correlated with an increased degree of atomic order in carbonate hydroxyapatite crystals, the latter determined using infrared spectroscopy. Here we present a reference infrared spectral library of tooth enamel from African ungulates, based on the grinding curve method, which serves as baseline to track the diagenetic history of carbonate hydroxyapatite in different species and thus select the best-preserved specimens for dating.

## Background & Summary

A large portion of the evolutionary history of the genus *Homo* and our species *Homo sapiens* is based on the Pleistocene (2.588-0.012 Ma) palaeontological record of Africa^1,2^. Our ability to understand diachronic change during this geological epoch relies on the application of dating methods that can provide absolute age determinations for human fossils. For periods older than ~50,000 years ago, which is the limit of radiocarbon dating, electron spin resonance coupled with uranium-series dating (ESR/U-series) of carbonate hydroxyapatite in tooth enamel is the only method that can provide accurate ages directly from fossils^3,4^.

ESR is a trapped-charge dating method based on the capacity of some minerals (e.g., carbonate hydroxyapatite and quartz) to store the energy coming from radiations emitted in the environment around the sample (i.e., produced from the radioactive decay in sediments and from cosmic rays) and in some cases in the sample itself (e.g., dental tissues that incorporate uranium during burial). The amount of energy absorbed by the sample, or “dose”, is a function of the radioactivity and the burial time. This allows using this phenomenon for dating: an age can be determined by dividing the dose absorbed by the sample (called the equivalent dose and measured in Gray, Gy) by the amount of energy emitted per year (the dose rate, measured in Gray per year, Gy·a^−1^). For fossil teeth, the enamel is targeted for ESR dating because it is the most mineralised dental tissue (~96% of hydroxyapatite in enamel versus ~70% in dentine) and the most crystalline. However, the uptake of trace elements such as uranium (U) in the dental tissues during burial in sediments, as a consequence of diagenesis, contributes to the dose rate and needs to be accounted for, hence the combination of ESR with uranium-series dating. U-series (^230^Th/U) is based on the radioactive decay of ^234^U to ^230^Th within the ^238^U decay chain^5,6^. A U-series age is obtained using the decay constants of the isotopes of interest as well as the ^234^U/^238^U and ^230^Th/^238^U ratios. The method is commonly used on speleothems, which incorporate U during the nucleation of the crystals (under the form of calcite or aragonite). In teeth, the application of U-series provides a minimum estimate in the best case, i.e., if no loss of U (“leaching”) occurred during burial, since the time between the burial of the tooth and the uptake of U is unknown. To overcome the difficulties related to the use of these two dating methods, it was proposed to combine ESR with U-series to reconstruct the U uptake mode in the dental tissues and derive the dose rate^7^. Nevertheless, U leaching remains a serious issue since part of the information needed to reconstruct the kinetics of U in the dental tissues is missing, hindering a precise reconstruction of the dose rate and hence the calculation of accurate ages. Ideally, one would date teeth with little U (i.e., a few hundred ppb) since such levels of U in the dental tissues do not impact significantly the dose rate^8^.

Depending on parameters such as the sedimentological context, the state of preservation, and the age of the sample, the U-content varies greatly from one site to the other, and even within the same site or sediment layer^9^. During burial in sediments and at pH values < 8, carbonate hydroxyapatite crystals in tooth enamel tend to become larger and more ordered at the atomic level according to Ostwald ripening, as a consequence of diagenetic processes involving the dissolution and reprecipitation of crystals. The latter entails the incorporation of uranium from sediment, as well as its leaching, and translates into an increased degree of atomic order, broadly defined here as crystallinity, in the carbonate hydroxyapatite crystals^10^. Therefore, characterising the integrity of enamel samples prior to dating is a fundamental step in order to select the best-preserved teeth, namely those with low U-content. Several studies have shown the effectiveness of Fourier transform infrared spectroscopy (FTIR) in determining the degree of atomic order of carbonate hydroxyapatite crystals in enamel, dentine, cementum, and bone using the grinding curve method in transmission mode^11–13^. This method allows decoupling the opposite effects of particle size and degree of atomic order on the shape of infrared spectra by plotting the full width at half maximum (FWHM) of the ν_3_ band of phosphates (1034 cm^−1^) and the infrared splitting factor (IRSF) of the ν_4_ band of phosphates (peaks at 604 and 565 cm^−1^) upon repeated grinding of the same potassium bromide (KBr) pellet. Using the grinding curves, it has recently been shown that an increase of atomic order is positively correlated with higher U-content (several ppm of U)^9,14^. The FTIR grinding curves thus represent a rapid pre-screening method to select the best-preserved samples, which are less likely to be affected by uranium leaching.

**Fig. 1:**
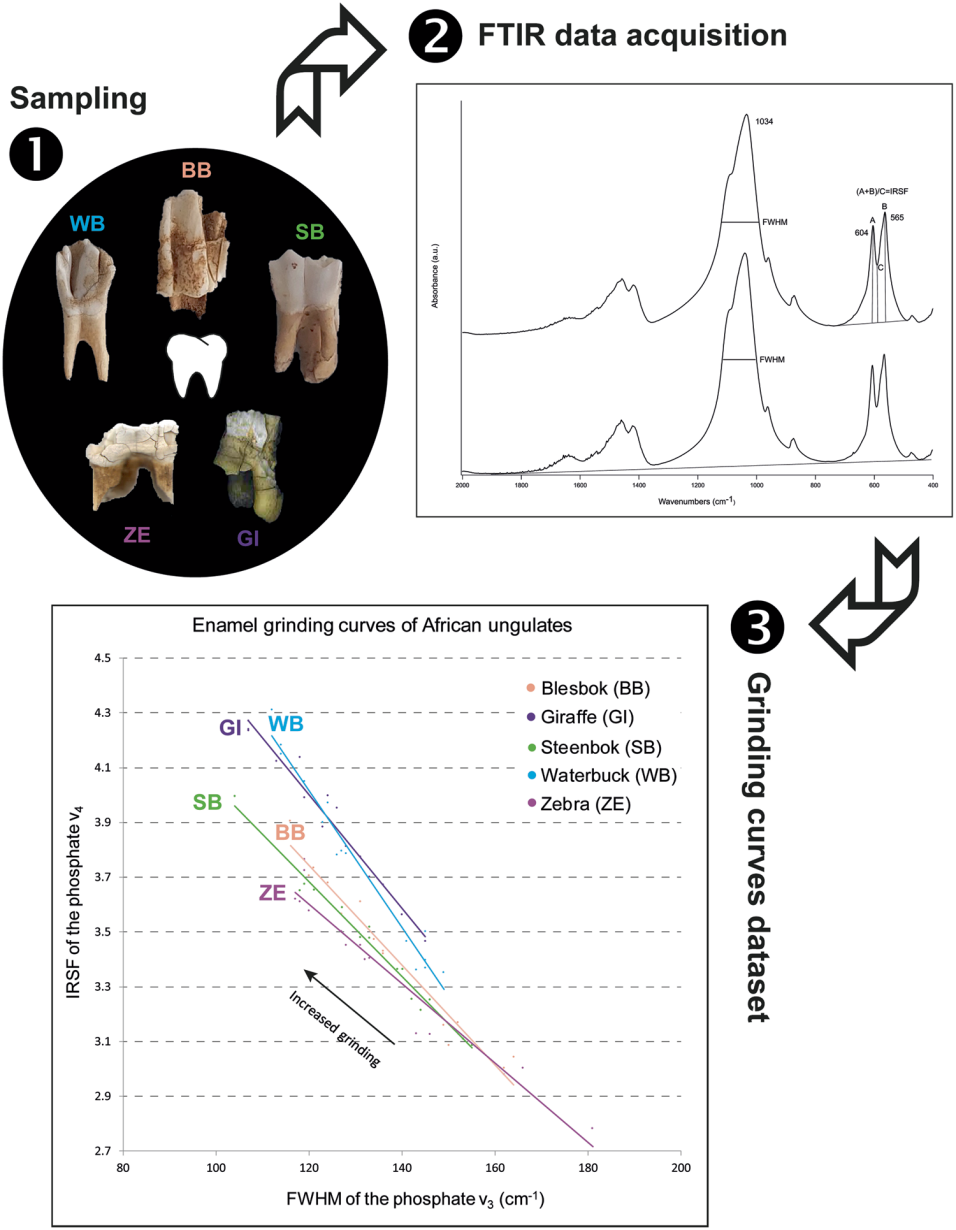
Workflow followed to compile the reference spectral library and grinding curve plot.

While teeth are partially destructed when applying standard protocols similar to the one used in this study, technical developments and methodological improvements allowed limiting the damages on human teeth to be dated using ESR/U-series^15–17^. However, considering the relative scarcity of human teeth in the palaeontological record and their value for palaeoanthropology, other mammals associated with human activities or with human presence in the landscape and at archaeological sites are usually targeted for ESR/U-series dating^8,18–25^. These include mainly ungulates, which occur in large populations across Africa and constitute the bulk of classification systems aimed at describing the evolutionary history of mammals (e.g., land mammal ages)^26,27^, and whose teeth exhibit thicker enamel. As shown in an earlier study^12^, the degree of crystallinity of carbonate hydroxyapatite in fresh tooth enamel changes based on the species, thus yielding different FTIR grinding curves. Therefore, in order to apply the grinding curve method to fossil ungulate teeth potentially suitable for ESR/U-series dating, a baseline should be created that accounts for the varying degrees of crystallinity in different ungulate species. Here we present a database of FTIR spectra of ungulate species from southern Africa that were used to create reference grinding curves to assess the crystallinity of fossil specimens of unknown state of preservation (Fig. 1). This information can be used to select the best-preserved specimens for ESR/U-series dating, thus minimising the risk of incurring in samples affected by uranium leaching. This database includes mainly bovids ranging from small to large body size, as well as species from other important families found in Pleistocene assemblages. The grinding curve plot shows clear differences in enamel carbonate hydroxyapatite crystallinity between species and across families. Our aim is to provide an open-access spectral library and grinding curve reference plot that can be used to improve the accuracy of ESR/U-series dates on ungulate teeth. The same dataset may find application also in the analysis of carbon and oxygen stable isotopes for palaeoenvironmental reconstructions, which may be affected by diagenetic processes.

## Methods

Teeth were obtained from the collection of modern vertebrates of the National Museum Bloemfontein (South Africa), stored at the Florisbad Quaternary Research Station. All specimens are permanent and fully erupted and derive from adult individuals that lived in South African populations. No selection was made based on tooth type or position in the oral cavity, since it was demonstrated that tooth enamel crystallinity is not affected by these parameters^12^. Similarly, it was demonstrated that there is no intra-species variability^12^. However, we focused on premolars and molars. Sample collection targeted the ungulate species that are more often found in the archaeological and palaeontological record and/or are the nearest extant parallel to extinct species within the same genus: for instance, *Antidorcas bondi* (extinct Bond’s springbok) and *Antidorcas marsupialis* (extant springbok); *Damaliscus niro* (extinct species of blesbok) and *Damaliscus pygargus* (extant blesbok); *Hippopotamus gorgops* (extinct giant hippopotamus) and *Hippopotamus amphibius* (extant hippopotamus)^9,14,28–31^. These are among the species that are more likely to be selected for ESR/U-series dating.

A total of 15 species were sampled, which are listed in Table 1. One tooth was selected for each species and one enamel fragment was removed from each tooth, which provided the powder necessary for FTIR analyses. Enamel was separated from dentine using a diamond saw and cleaned using a rotary drill bit coated with diamond grit. Enamel fragments were crushed in an agate mortar and pestle and the resulting powder was sieved through a 200 µm mesh to obtain a homogeneous particle size.

**Table 1:** List of the species analysed in this study (P: premolar; M: molar).

Common name	Species	Subfamily	Family	ID	Position	Locality
Plains zebra	*Equus quagga*	Equinae	Equidae	NMBF 382	P_2_ right	Unknown
Southern white rhinoceros	*Ceratotherium simum*	Rhinocerotinae	Rhinocerotidae	NMBF 624	P^1^ left	Zululand
Giraffe	*Giraffa camelopardalis*	Giraffinae	Giraffidae	NMBF 6062	P^3^ right	Unknown
Hippopotamus	*Hippopotamus amphibius*	Hippopotaminae	Hippopotamidae	NMBF 335	P^2^ right	Unknown
Waterbuck	*Kobus ellipsiprymnus*	Reduncinae	Bovidae	NMBF 8893	P_1_ right	Kruger National Park
Southern reedbuck	*Redunca arundinum*	Reduncinae	Bovidae	NMBF 8905	M_3_ left	Unknown
Impala	*Aepyceros melampus*	Aepycerotinae	Bovidae	NMBF 8814	P_1_ left	Unknown
Greater kudu	*Tragelaphus strepsiceros*	Bovinae	Bovidae	NMBF 236	P^1^ right	Unknown
Common eland	*Taurotragus oryx*	Bovinae	Bovidae	NMBF 8756	M^1^ left	Bloemfontein Zoo
Springbok	*Antidorcas marsupialis*	Antilopinae	Bovidae	NMBF 103	P^2^ left	Unknown
Steenbok	*Raphicerus campestris*	Antilopinae	Bovidae	NMBF 9343	M^1^ left	Bultfontein Road
Black wildebeest	*Connochaetes gnou*	Alcelaphinae	Bovidae	NMBF 8707	M^3^ right	Willem Pretorius Game Reserve
Blue wildebeest	*Connochaetes taurinus*	Alcelaphinae	Bovidae	NMBF 52	M^3^ right	Unknown
Hartebeest	*Alcelaphus buselaphus*	Alcelaphinae	Bovidae	NMBF 12421	M_2_ right	Soetdoring Nature Reserve
Blesbok	*Damaliscus pygargus*	Alcelaphinae	Bovidae	NMBF 9384	P^1^ left	Erfenisdam, Theunissen

FTIR spectra were acquired using the grinding curve method. About 5 mg of sample powder and 20 mg of FTIR-grade KBr (Sigma-Aldrich) were placed in an agate mortar and pestle and lightly ground. The mixture was transferred to a pellet die and pressed by applying 2 tons using a benchtop mini-press (Specac). The pellet was then analysed in transmission mode using a Thermo Scientific Nicolet iS5 infrared spectrometer equipped with an iD1 transmission module and a deuterated triglycine sulphate (DTGS) detector. Spectra were collected in the 4000-400 cm^−1^ spectral region at 4 cm^−1^ resolution and in 32 scans, and processed using OMNIC v. 9.13. The FWHM of the phosphate ν_3_ band (1034 cm^−1^) and the IRSF of the phosphate ν_4_ band (604 and 565 cm^−1^) in carbonate hydroxyapatite were calculated using Macros Basic v. 10 and according to standard literature^12,32^. The baseline for the calculation of the ν_3_ intensity was drawn between 2000 and 400 cm^−1^, whereas the baseline for the calculation of the intensity of the two peaks in the ν_4_ band was drawn between 820 and 400 cm^−1^. After processing the spectrum, the pellet was removed from the instrument and half of it was ground more vigorously in order to reduce particle size. The other half was discarded prior to grinding to avoid overloading of the infrared spectrum caused by increased absorption promoted by the larger overall surface area of the particles. After adding 10 mg of KBr, a second pellet was prepared and analysed following the procedure described above. The same operation was repeated a third time by grinding half of the second pellet with even more strength. The FWHM and IRSF values of carbonate hydroxyapatite for each grinding were plotted to obtain a trendline, or grinding curve. Four additional replicates were produced using enamel powder from the same tooth, for a total of five grinding curves for each ungulate species. Considering the subjective nature of the grinding process, the same operator should perform all of the grindings of a given curve.

## Data Records

**Fig. 2:**
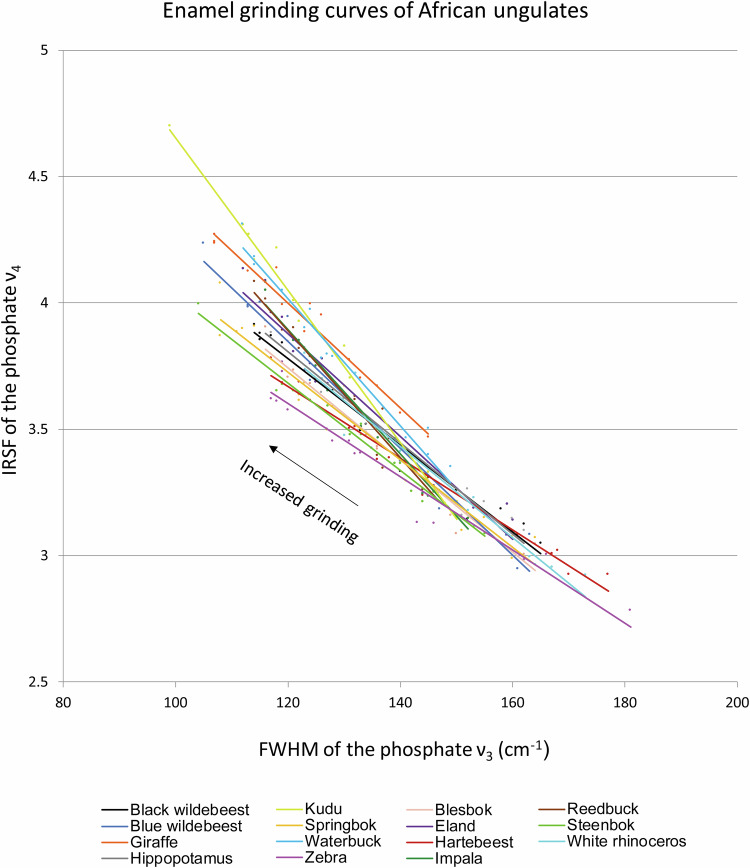
Grinding curve plot including all the species in the database. Note the large range of offsets among curves.

The infrared spectra used for establishing grinding curves are located in an online repository (“Enamel_African_ungulates.zip”)^33^ in a folder of raw spectra (“FTIR spectra”) together with a separate file including the FWHM and IRSF values for each spectrum and a plot showing the grinding curves of each ungulate species (“Enamel_grinding_curves.xslx”) (Fig. 2), and the macro file used to calculate the FWHM and IRSF values (“IRSF_FWHM.mac”). The library includes 225 infrared spectra in “.spa” and “.csv” formats (total: 450 files), which can be read by spectroscopy programmes. Spectra are named with progressive numbers in order to distinguish replicates and number of grindings; for instance, “Giraffe2_03” indicates the third grinding of the second grinding curve of giraffe enamel. The same naming system is followed in the grinding curve file.

## Technical Validation

At the spectroscopic level, the grinding curve method allows detecting differences in atomic order in different samples independently from particle size, since FWHM and IRSF are calculated over a range of particle sizes. This makes it unnecessary to standardise the initial amount of sample and KBr, the degree of grinding, and the amount of pressure applied to the mixture to obtain a pellet. While we used a press equipped with a gauge that provides a measure of the applied pressure, equivalent results can be achieved with a hand press; in both cases the same operator should perform all of the grindings of a given curve to ensure that each successive grinding is performed more vigorously than the previous one.

An earlier study^12^ on human, horse, cow, and dog enamel used the least squares method to fit the grinding curve data, showing that the correlation coefficient (R^2^) of FWHM and IRSF is always above 0.9. R^2^ values above 0.9 can be observed in the library presented herein (Table 2), thus validating the technical quality of the grinding curves. Similar results have been obtained by applying the grinding curve method to calcite^34^ and aragonite^35^.

**Table 2:** Correlation coefficient (R^2^) of the enamel grinding curves for each species.

Species	R^2^
*Equus quagga*	0.94
*Ceratotherium simum*	0.95
*Giraffa camelopardalis*	0.97
*Hippopotamus amphibius*	0.96
*Kobus ellipsiprymnus*	0.97
*Redunca arundinum*	0.95
*Aepyceros melampus*	0.97
*Tragelaphus strepsiceros*	0.98
*Taurotragus oryx*	0.95
*Antidorcas marsupialis*	0.96
*Raphicerus campestris*	0.98
*Connochaetes gnou*	0.97
*Connochaetes taurinus*	0.97
*Alcelaphus buselaphus*	0.98
*Damaliscus pygargus*	0.96

**Fig. 3:**
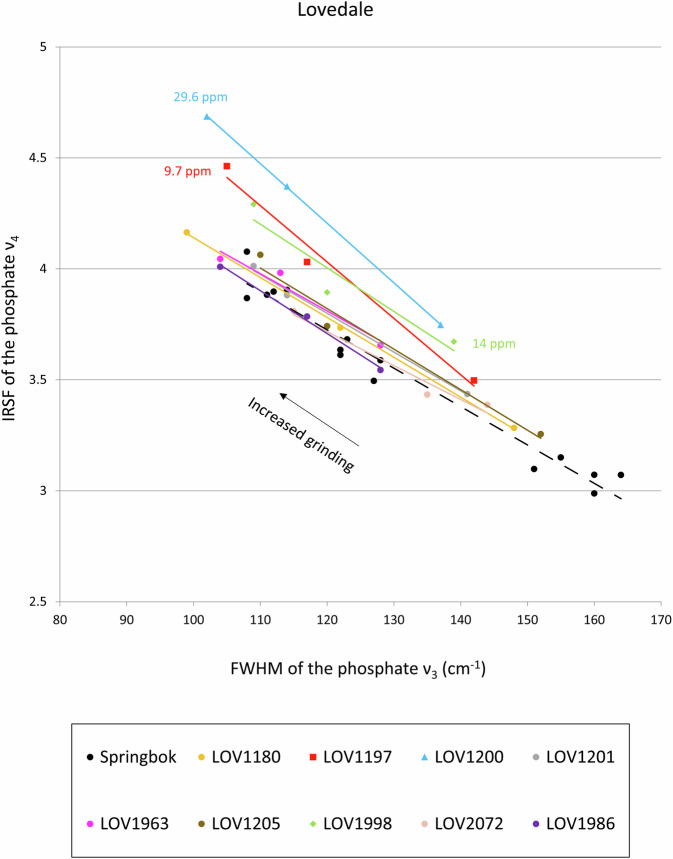
Grinding curves of fossil teeth from Lovedale (South Africa) compared to a reference curve of springbok (dashed line). The three most recrystallised specimens, which exhibit greater curve offset compared to the springbok curve, are also the richest in uranium (ranging from 9.7 to 29.6 ppm). All other specimens are characterised by amounts of uranium ranging from 1.7 to 5.8 ppm (data from Richard *et al*.^9,14^).

Recent studies have used the grinding curve of present-day *A. marsupialis* enamel as reference for fossil teeth of small-sized Antilopinae, most likely the extinct species *A. bondi*, recovered at the Late Pleistocene archaeological site of Lovedale (South Africa), dated to 77–56 ka^9,14,36^. The enamel in the fossil specimens showed varying degrees of crystallinity, based on the offset of the grinding curves with the reference curve of *A. marsupialis*, which positively correlated with the amount of uranium incorporated as a result of diagenetic alteration during burial in sediments. The most crystalline samples, i.e. the samples exhibiting the largest offset compared to the reference curve, were also the richest in uranium, whereas samples with intermediate uranium contents exhibited grinding curves located between modern reference and extensively recrystallised specimens (Fig. 3). Therefore, the grinding curve method of tooth enamel can be used as a screening tool to select the least crystalline (i.e., best-preserved) specimens for ESR/U-series dating, and thus avoid materials with high U-content or that suffered from uranium leaching.

## Usage Notes

This reference library of infrared spectra and related grinding curve plot can be used to determine the degree of crystallinity of enamel carbonate hydroxyapatite in teeth of unknown state of preservation from the same species analysed in this study. The plot will be most useful with regard to the African continent, but may provide support also with closely related species from Asia and Europe (e.g., other taxa within the Bovinae subfamily, Bovini tribe). The chart can be used to display only specific taxa, which are relevant to the research question. To determine the crystallinity of a tooth of unknown preservation, users should develop a grinding curve by plotting the FWHM and IRSF values of each FTIR spectrum obtained from the enamel. The offset between this curve and the reference curve will provide an assessment of the degree of crystallinity of the tooth. If the curves overlap, the fossil tooth is well preserved. If an offset exists, then the fossil tooth underwent diagenetic alteration and should not have priority for ESR/U-series dating.

This method will yield best results if applied systematically to all the teeth from a given assemblage (e.g., same fossil/archaeological site) in order to track diagenetic processes in three dimensions throughout the depositional contexts, especially when paired with the characterisation of the sedimentary matrix by FTIR spectroscopy^37–40^. FTIR micro-spectroscopy^41,42^, Raman hyperspectral imaging^43^, and laser-induced fluorescence^9^ may provide additional information about the state of preservation of the specimens and help select specific regions of interest in the enamel. Obviously, adding more species to the library will help expand the references and make comparisons with unknown samples more accurate. In addition, the library may be used to assess the crystallinity of enamel in relation to the analysis of carbon and oxygen stable isotopes, which are important parameters in palaeoecological studies^44^. While there are no studies linking diagenetic alteration as determined with FTIR grinding curves with significant deviations in δ^13^C and δ^18^O values, the fact that the uranium content of enamel undergoes important changes with diagenesis may imply that deviations are large also for carbon and oxygen, which are among the main components of carbonate hydroxyapatite. Scanning electron microscopy combined with FTIR micro-spectroscopy and electron microprobe analysis of fossil teeth characterised by extensive recrystallisation seem to point in this direction^42^.

## Data Availability

The macro used to calculate the FWHM of the ν_3_ band and the IRSF of the ν_4_ band of phosphates runs in Macros Basic coupled with OMNIC (including versions of the software older than the one used in this study) and it is available in the online repository^33^ with the raw spectra (“IRSF_FWHM.mac”). Given the simple nature of the calculations, similar macros can be easily developed using different spectroscopy programmes.
